# A cluster randomized web-based intervention trial to reduce food neophobia and promote healthy diets among one-year-old children in kindergarten: study protocol

**DOI:** 10.1186/s12887-018-1206-8

**Published:** 2018-07-14

**Authors:** Eli Anne Myrvoll Blomkvist, Sissel Heidi Helland, Elisabet Rudjord Hillesund, Nina Cecilie Øverby

**Affiliations:** Faculty of Health and Sport Sciences, Department of Public Health, Sport and Nutrition, University of Agder, Kristiansand, Norway

**Keywords:** Children, Kindergarten, Food neophobia, Diet variety, Parental feeding practices, Cognitive development, Overweight, Sapere method, Sensory education

## Abstract

**Background:**

A child’s first years of life are crucial for cognitive development and future health. Studies show that a varied diet with a high intake of vegetables is positive for both weight and cognitive development. The present low intake of vegetables in children’s diets is therefore a concern. Food neophobia can be a barrier for vegetable intake in children. Our hypothesis is that interventions that can increase children’s intake of vegetables should be introduced early in life to overcome children’s neophobia. This study aims to develop, measure and compare the effect of two different interventions among one-year-old children in kindergartens to reduce food neophobia and promote healthy diets.

**Methods:**

The kindergartens are randomized to one of three groups: two different intervention groups and one control group. We aimed to include a total of 210 children in the study. The first intervention group will be served a warm lunch meal with a variety of vegetables, 3 days a week during the intervention period of 3 months. The second intervention group will be served the same meals and, in addition, kindergarten staff will be asked to implement pedagogical tools including sensory lessons, adapted from the Sapere method, and advices on meal practice and feeding practices. The control group continues their usual meal practices. Parents and kindergarten staff will complete questionnaires regarding food neophobia, food habits and cognitive development at baseline and post intervention. A similar intervention among 2-year-old children in kindergarten has been implemented and evaluated earlier. We will investigate whether a digital version of this intervention has an effect, because digital interventions can be easily implemented nationwide. We will also investigate whether there are benefits of conducting such interventions in younger children, before the onset of food neophobia. Questionnaires, information videos and recipes will be digitally distributed.

**Discussion:**

Results of this study will provide new knowledge about whether a sensory education and a healthy meal intervention targeting children, kindergarten staff and parents will reduce levels of food neophobia in children, improve parental and kindergarten feeding practices, improve children’s dietary variety, improve children’s cognitive development and reduce childhood overweight.

**Trial registration:**

ISRCTN98064772.

## Background

What we eat has significant impact on health and disease [1]. In Norway, eating an unhealthy diet is the second most important risk factor for disease burden [2]. A low intake of fruits and vegetables and a high intake of energy dense foods increases the risk for non-communicable diseases [1, 3, 4]. To reduce this risk The World Health Organization (WHO) recommends an increased intake of fruit and vegetables throughout the world [5]. The increasing prevalence of obesity among children is a global health challenge [6, 7]. Although an inverse relationship between fruit and vegetable intake and obesity in children remains somewhat unclear [8], a healthy dietary pattern with a high intake of fruit and vegetables is crucial for health and development. Studies have also shown that diet has an impact on children’s cognitive development [9], and that healthy dietary patterns in childhood can influence cognitive and neuropsychological outcomes [10, 11]. The World Health Organization (WHO) has stated that proper nutrition during the 1000 days between a woman’s pregnancy and her child’s 2nd birthday (the 1000 day window) has a profound impact on a child’s ability to grow, learn and thrive, and hence a lasting effect on a country’s health and prosperity [12].

In Norway the average intake of fruits and vegetables in one-year old children is only half of the recommended intake [13]. A low intake of vegetables is particularly challenging regarding health. A national survey found that one-year old children ate only 32 g of vegetables per day on average [13] . One barrier for vegetable intake in children is food neophobia, meaning a reluctance to taste and eat new foods. This trait is most explicit in children between 2 and 6 years of age [14]. Food neophobia is associated with a low intake of vegetables and a poorer dietary quality [15, 16]. Helland et al. [17] found that food neophobia was negatively associated with intake of fruit and vegetables, berries and fish in two-year olds. Moding and Stifter [18] suggest that rejection of new foods during infancy predicts neophobia during early childhood. Fletcher et al. [19] found that an early liking for fruit and vegetables predicted increased later intake, so they hypothesize that increasing early exposure to fruit and vegetables may have long-term beneficial consequences.

Food neophobia and scepticism to eat new foods is modifiable. Several intervention studies have shown that repeated exposure, where pre-school children are exposed to either vegetables alone or to vegetables combined with other flavours, for instance a dip or sauce, can increase children’s willingness to taste and eat vegetables [20–24]. Researchers have also found that hiding vegetables in mixed courses can be an effective strategy to increase children’s vegetable intake [25]. Role modeling is a well-known strategy that can influence food intake in children [26–29]. Social Cognitive Theory suggests that modelling by teachers or by peers, would be one of the most effective methods to encourage food acceptance in preschool children [30]. Hendy et al. [26] found that enthusiastic teacher modelling was more effective than silent teacher modelling, and that peer modelling was the most effective method to encourage new food acceptance in preschool children.

Another area of research is sensory education, allowing children to explore foods with their senses by smelling, touching, hearing, watching and tasting. The aim of sensory training is to increase the willingness to taste new foods and thereby increase intake of vegetables or other foods in children [31–34]. The Sapere method based on Puisais’ work *Le Goût de L’enfant* [35] can be one way of learning about food through senses and language in kindergartens and schools. The sensory-based food education programme, which originated in France, has since been translated to Swedish [36] and is being used both in schools and kindergartens in Sweden [37] To our knowledge, the Sapere method has not been subject to research in preschoolers in Norway except from the study done by our research group [38]. Helland et al. [17, 38] have tested the Sapere sensory education in toddlers between the ages of two and 3 years. We will now investigate whether there are benefits of conducting such interventions in younger children, before the usual onset of food neophobia.

Toddlers in Norway spend much of their time in kindergarten and more than 80% of all children between 1 and 2 years of age attend kindergarten [39]. The recent (2017) Framework plan for kindergartens [40] suggests that kindergarten staff use mealtimes and cooking to enable the children to enjoy food, participate, communicate and feel togetherness. Food and feeding practices in kindergarten can influence children’s diet and eating habits [41], and kindergarten staff have a great responsibility and opportunity when it comes to teaching children about food and meals. The kindergarten setting is an arena where both repeated exposure to new foods and sensory education can be implemented systematically, as well as an arena where the importance of caregivers as role models can be explored.

The Internet plays an important role in our everyday lives. A recent review found that caregivers use the internet for both information, support and education [42]. An earlier study in seven European countries found that 71% of Internet users had used the Internet for health purposes [43]. It is reasonable to believe that the proportion is even higher today. A recent study showed that providing kindergarten and elementary school educators with web-based resource materials improved their attitudes, increased their knowledge and lead to positive behavioural intentions concerning educating their students about oral health [44]. We believe that this can be applied to other health concerns as well.

The aim of the present study is to develop and evaluate the effect of two different interventions among one-year-old children in kindergartens in four counties in Norway. The interventions aims to promote a healthy and varied diet in young children that can facilitate cognitive development and help to prevent future overweight.

### Outcomes

Primary outcomes:

1. Child vegetable intake assessed at baseline, after the intervention, and at the ages of 36 and 48 months.
2. Children’s level of food neophobia assessed at baseline, after the intervention, and at the ages of 36 and 48 months.
3. Child dietary habits and food variety assessed at baseline, after the intervention, and at the ages of 36 and 48 months.

Secondary outcomes:

4. Child cognitive development assessed at baseline, after the intervention, and at the ages of 36 and 48 months.
5. Self-reported weight and height assessed at baseline, and at the ages of 36 and 48 months.
6. Parental and kindergarten staff feeding practices assessed at baseline, after the intervention, and at the ages of 36 and 48 months.

## Methods

### Study design

This study is a cluster randomized controlled trial. It is an ongoing study.

The kindergartens are randomized to one of three groups: two different intervention groups and one control group. We aimed to include 210 children in the study.

A similar intervention among 2-year-old children in kindergarten has been implemented and evaluated earlier [38] and we will now investigate the effect of a digital version of such an intervention, because a digital intervention can be more easily implemented into kindergarten daily life. Information videos and recipes for the project will be included in a password protected study web page and questionnaires will be distributed by e-mail.

The protocol for the present study was approved by the Norwegian Centre for Research Data (ref.nr 49951). Informed consent was obtained from the kindergarten manager and from one of the parents of all participating children when registering for the study.

### Recruitment and participants

**Fig. 1:**
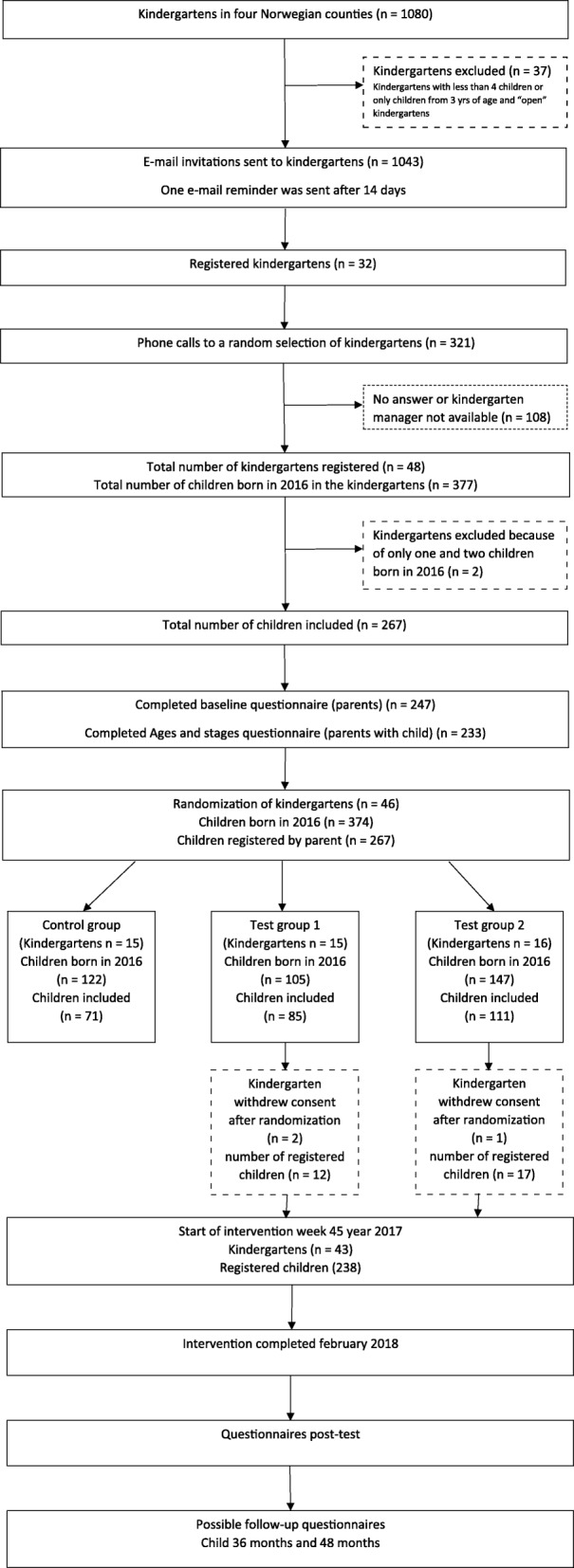
Flowchart of the study design

The kindergartens were recruited from four counties in Norway; Telemark, Oppland, Sør-Trøndelag and Møre og Romsdal. An invitation to participate was first sent by e-mail to the managers of kindergartens in the two counties Telemark and Oppland and due to low participation, two new counties were included: Sør-Trøndelag and Møre og Romsdal. The invitations were sent to kindergartens registered at The Norwegian Directorate for Education and Training (UDIR) (*n* = 1080). Kindergartens registered as “open” kindergartens where children and their parents attend together (*n* = 18), kindergartens registered with less than 4 children (*n* = 7) and kindergartens with children from 3 to 5 years only (*n* = 12) were not invited to participate (Fig. 1). The invitation included detailed information about the study and a link to the study registration web page. A reminder e-mail was sent to the kindergartens 2 weeks after the first e-mail. Because few kindergartens (*n* = 32) registered for the study initially, a random selection of kindergartens in all four counties was additionally contacted by phone (*n* = 321). A total of 48 kindergartens registered for the study (Fig. 1). Two of the kindergartens withdrew before randomization because they had fewer than three children born in 2016 (Fig. 1).

The pedagogical leaders in the participating kindergarten departments were asked to distribute an electronic invitation letter to the parents providing information about the study and a link to the registration web page where parents could register their child to participate in the study. Inclusion criteria for the children participating in the study was that they had to be born in the year of 2016 and that at least one of the parents could read and understand Norwegian. A total of 267 children were registered for the study (Fig. 1).

### Intervention

The participating kindergartens (*n* = 46) were randomized into two different intervention groups and one control group. Children in the first intervention group will be served a warm lunch meal with a variety of vegetables, 3 days a week during the intervention period that will last for 3 months. After 3 weeks with the first menu there will be a one-week break before starting the serving of meals from the second menu 3 days a week in three more weeks and after another one-week break, 3 weeks with the third and last menu. The kindergartens will have access to a total of nine different recipes in a password protected web page especially designed for each intervention group. (Table 1) Each of the three menues has one “focus” vegetable, i.e. spinach, celeriac and fennel. A minimum of two meals per week will include the focus vegetable so that the children are exposed to each vegetable at least six times during the menu period of 3 weeks (Table 1).

**Table 1:** Lunch dishes cooked in the intervention kindergartens

	Vegetarian	Fish	Vegetarian
Menu 1 spinach	Pasta with vegetables and feta cheese (includes spinach)	Pan fried fish with carrot purée	Spinach and lentils soup
Menu 2 celeriac	Celeriac soup	Salmon with celeriac purée	Vegetable stew (includes celeriac)
Menu 3 fennel	Minestrone soup (includes fennel)	Fish cakes with oven baked vegetables (includes fennel)	Potato and broccoli omelet

Children in the second intervention group will be served the same meals from the same menus as described for intervention group 1. In addition the kindergarten staff in intervention group 2 will be asked to implement pedagogical tools including i) weekly sensory lessons (Sapere method) [35] for the participating children and ii) advice on meal practice and feeding practices during mealtime. Children participating in the sensory lessons will have three more exposures, a total of at least nine exposures, of the selected “focus” vegetables.

Meal practice and Feeding practices recommendations are presented in short information videos on the study web page which is only available for the second intervention group. The videos contain information about food neophobia, repeated exposure, role modeling, our five senses, basic tastes, and the Sapere method.

The control group will continue their usual meal practices.

### Measurement instruments

To evaluate the effect of the interventions on the given outcomes, parents and kindergarten staff will complete questionnaires at baseline and post intervention. There will be follow-up-questionnaires when the children are 36 and 48 months old.

A main questionnaire to the parents including all the outcome variables has been developed specifically for this study, except measures of cognitive development which is measured with the Ages and Stages Questionnaire (ASQ) [45]. A separate questionnaire was developed for the pedagogical leaders in the participating departments. All measurements are described in detail below.

#### Measures of child food neophobia

Child food neophobia is measured with a 6-item version of Pliner’s 10-item Child Food Neophobia Scale (CFNS) [46]. The Child Food Neophobia scale (CFNS) is a validated tool which uses parental reporting of child neophobia. The 6-item version of CFNS is commonly used to measure food neophobia in young children and has been used with children as young as 2 years [15, 17, 47, 48]. Responses are ranged from “strongly disagree” to “strongly agree” on a seven-point scale. The CFNS items have been translated from English into Norwegian, and back-translated into English by members of our research group earlier [17]. The CFNS was included in the parental questionnaire.

#### Measures of parental and kindergarten staff feeding practices

Parental and kindergarten staff feeding practices is measured with the Comprehensive Feeding Practices Questionnaire (CFPQ), which has been validated earlier [49]. CFPQ has been used to assess parental feeding practices at 18 months [50], and has already been translated to Norwegian and validated in parents of 10-to-12-year-olds [51].

The original CFPQ includes 12 subscales. The following eight subscales are included in the parental questionnaire when the child is 1 year old: Child control, Emotion regulation, Encourage balance and variety, Environment, Food as reward, Modeling, Pressure and Restriction for health. The four other subscales: Involvement, Monitoring, Restriction for weight control and Teaching about nutrition will be included in the parental questionnaire to be used when the children have reached the age of three and 4 years.

Kindergarten staff will complete a modified version of the CFPQ, adapted to a kindergarten context. The following seven subscales were included in the questionnaire to the pedagogical leaders: Child control, Emotion regulation, Encourage balance and variety, Food as reward, Modeling, Pressure and Restriction for health.

#### Measures of children’s food intake, food variety and vegetable liking

Child food intake and food variety is measured by selected items from a food frequency questionnaire that has been validated and used in large national surveys [13]. Amounts of food is not measured, only frequencies of intake. Questions on how often the child eats fruits, berries, vegetables and potatoes are included, in addition to questions about bread and cereals, drinks, warm meals and snacks. The response options for intake of fruits and vegetables are: never, < 1/month, 1–3/month, 1–2/week, 3–4/week, 5–6/week, 1/day, 2/day, > 3/day. In addition to these food frequency questions, questions about duration of breastfeeding and time of introduction to solids are also included.

Measure of vegetable liking is adapted from a questionnaire used in the Australian study Nourish [52]. The answers are graded as 1: likes a lot, 2: likes a little, 3: neither likes or dislikes, 4: dislikes a little, 5: dislikes a lot, 6: never tried.

#### Measures of food refusal and food fussiness

Questions about child food refusal and food fussiness were adapted from The Nourish study questionnaires for children at the age of 14 months and 2 years [52]. Questions were translated into Norwegian by the author and back-translated by two co-authors to ensure that the meaning of the questions remained the same as in the original questionnaire.

#### Measures of weight and height

Measures of weight and height are self-reported. Parents are asked to report child weight and height in the most recently health control from the children’s health card.

#### Measures of other variables

Food frequency questions about parental intake of fruits, berries and vegetables, as well as questions about parental age, height and weight, ethnicity, length of education and occupation are also included in the questionnaire.

Level of food neophobia in parents and kindergarten staff is measured with the original 10-item version of the FNS [53].

Questions about the kindergartens meal routines and food serving are included in the questionnaire to the pedagogical leaders.

#### Measures of cognitive development

Children’s cognitive development is measured with the Ages and Stages Questionnaire [45]. This questionnaire has been widely used in both clinical and research settings in several countries [54, 55]. It consists of 19 different questionnaires covering the age-range of 4 to 60 months. The questionnaires cover five different domains: communication, gross motor, fine motor, problem solving and personal social skills. The Norwegian version of ASQ has also been validated [56].

#### Compliance with intervention elements

The pedagogical leaders in the two intervention groups will complete a weekly short evaluation form on the study web page. They are asked to assess the success of the implementation of the intervention elements on a scale of zero to ten and to describe whether there are discrepancies from the project plan as described in the study web page.

### Sample size calculation

Sample size was calculated according to the primary outcome food neophobia. A previous cross-sectional trial of 505 toddlers in Southern Norway [17] resulted in a mean neophobian score of 18.2 (SD:9.3). We assumed that a mean score reduction in the level of food neophobia from 18.2 to 12.0 would be of public health value. Using a power of 80% and type 1 error of 5%, this suggested 36 participants were needed in each group. To adjust for within cluster variation we assume an intra-cluster correlation coefficient of 0,1 and a design effect factor of 1,6 expecting 7 participants in each cluster [57]. Based on these calculations we would need 58 participants in each group. Due to a probable loss of participants of 20%, we aimed to recruit 70 children in each of the three groups, a total of 210 children in this study.

### Randomization

The 46 registered kindergartens were randomized from a block of 48 into three groups.

### Data analysis

Data will be analyzed when the data collection is completed during springtime 2018.

Our primary goals are to detect differences in food neophobia scores, vegetable intake and food variety between each of the intervention groups and the control group.

## Discussion

Children today spend a large amount of time in Kindergarten. Kindergartens are a potentially important setting for influencing children’s food choice at an early age and there has been a call for intervention studies in this field [58]. With this study we are investigating the effectiveness of a web-based multi-component intervention in kindergarten. We have developed a web-based intervention that may easily be implemented in kindergartens throughout the country. The intervention kit includes three elements: a pedagogical tool (the Sapere method), a menu of associated lunch dishes and information videos targeting kindergarten staff and parents.

The strengths of our study are that it is being conducted in a natural setting, making it possible to reproduce in other kindergartens if it shows an effect. The Sapere method is widely used in some countries; however, few studies have evaluated its effect on children’s diet and health [35]. Further, distributing all study information electronically increases the availability of the intervention, making it easy for kindergarten staff and parents to find and use the recipes and tools. It may also be easier to track the children’s parents for follow-up-studies since the questionnaires are distributed by e-mail. To our knowledge there are few, if any, intervention studies on child food neophobia that has targeted children before the onset of neophobia, normally around the age of 2 years. In addition to investigating methods to reduce child food neophobia and increase child dietary variety, we also investigate if a dietary intervention in kindergarten can improve children’s cognitive development.

However, our study also has limitations. Recruitment of kindergartens and parents turned out to be quite difficult. It was also quite challenging to distribute the ASQ because there are different questionnaires for different ages (in months), and the registered children varied in age between 10 months and 20 months. The results of the study are based on parent-reporting which may have its weaknesses.

## Conclusion

Results of this study will provide new knowledge about whether a sensory education and a healthy meal intervention targeting children, kindergarten staff and parents will reduce levels of food neophobia in young children, improve parental and kindergarten feeding practices, improve children’s dietary variety, improve children’s cognitive development and reduce childhood overweight. This study will also provide knowledge about whether an electronically distributed intervention could be easily implemented in kindergartens nationwide.

## Abbreviations

ASQ: Ages and stages questionnaire
CFNS: Child food neophobia scale
CFPQ: Comprehensive feeding practices questionnaire
FNS: Food neophobia scale
